# MICA-129Met/Val as a therapeutic compass in idiopathic pulmonary fibrosis: prognosis and antifibrotic benefit

**DOI:** 10.3389/fimmu.2026.1722213

**Published:** 2026-07-20

**Authors:** Stefano Mocci, Caterina Mereu, Roberto Littera, Silvia Deidda, Federica Cannas, Michela Lorrai, Elena Zedda, Michela Murgia, Celeste Sanna, Erika Giuressi, Sara Lai, Andrea Perra, Matteo Floris, Sabrina Giglio

**Affiliations:** 1 Medical Genetics, Department of Medical Sciences and Public Health, University of Cagliari, Cagliari, Italy; 2 Centre for Research University Services (CeSAR, Centro Servizi di Ateneo per la Ricerca), University of Cagliari, Cagliari, Italy; 3 Medical Genetics, R. Binaghi Hospital, Cagliari, Italy; 4 AART-ODV (Association for the Advancement of Research on Transplantation), Cagliari, Italy; 5 Pneumology Unit, R. Binaghi Hospital, Cagliari, Italy; 6 Oncology and Molecular Pathology Unit, Department of Biomedical Sciences, University of Cagliari, Cagliari, Italy; 7 Department of Biomedical Sciences, University of Sassari, Sassari, Sardinia, Italy

**Keywords:** antifibrotic therapy, idiopathic pulmonary fibrosis, Met129Val, MICA, nintedanib

## Abstract

**Background:**

Idiopathic pulmonary fibrosis (IPF) is a progressive interstitial lung disease characterized by aberrant wound healing, immune dysregulation, heterogeneous trajectories, and poor prognosis. Only two antifibrotic agents, *nintedanib* and *pirfenidone*, are currently approved to slow disease progression, yet inter-individual variability in treatment benefit remains largely unexplained. In IPF, impaired NK-cell activity within the altered lung microenvironment may hinder removal of stressed or senescent cells, promoting fibrosis. The MHC-class I chain-related gene A (*MICA*) encodes a stress-induced ligand of the NKG2D receptor on NK and CD8^+^ T cells, crucial for immune surveillance.

**Methods:**

We analyzed clinical and genetic data from 129 Sardinian IPF patients genotyped for MICA and stratified by antifibrotic therapy (*nintedanib, pirfenidone*, no therapy, and switchers). Genotypes were assessed in relation to longitudinal lung function, overall survival (OS), and treatment-specific outcomes using multivariate Cox models adjusted for demographic and immunogenetic variables.

**Results:**

*MICA-129* genotype frequencies were comparable across treatment groups. In *nintedanib*-treated patients, the Val/Val genotype was associated with significantly reduced 48-month OS versus Met/Met and Met/Val (p = 0.005). No statistically significant association was observed among pirfenidone-treated patients. After adjustment, Val/Val remained an independent predictor of mortality in the *nintedanib* group (p = 0.01), irrespective of *HLA* background.

**Conclusions:**

The *MICA*-129 Val/Val genotype may represent a candidate immunogenetic marker associated with poorer outcome in *nintedanib*-treated patients with IPF, underscoring the influence of immune-genetic context on antifibrotic response. Although subgroup sizes limit precision, the consistency and mechanistic plausibility of the association support integrating *MICA* genotyping into future randomized trials to refine personalized therapeutic strategies in IPF.

## 1 Introduction

Idiopathic Pulmonary Fibrosis (IPF) is a chronic and progressive disorder characterized by the replacement of normal, elastin-rich pulmonary parenchyma with fibrotic tissue mainly composed of fibrillar collagen (1). Although the exact etiology of the disease remains unknown (2), emerging genetic evidence indicates that variants in telomere-maintenance genes (*TERT, TERC, RTEL1*) and surfactant protein genes (*SFTPC, SFTPA1/2, ABCA3*) significantly contribute to IPF susceptibility.

However, these established IPF susceptibility genes do not fully explain the marked interindividual variability in disease progression or response to antifibrotic therapy. In this context, immune-regulatory genes may represent biologically relevant candidates for understanding outcome heterogeneity.

To date, no therapeutic approach has demonstrated efficacy in achieving functional and lung recovery (3–5). For this reason, monitoring the forced vital capacity (FVC) and diffusing capacity of the lung for carbon monoxide (DLCO) is essential not only for assessing disease severity and progression but also for guiding the implementation of supportive therapeutic strategies such as oxygen supplementation, pulmonary rehabilitation, antifibrotic treatment, and, in the most severe cases, lung transplantation (3–5). Currently, the Food and Drug Administration and European Medicines Agency have approved two pharmacological drugs for treating IPF: *pirfenidone* and *nintedanib*, both of which act as antifibrotic agents (6). *Pirfenidone* is a synthetic pyridone compound with both antifibrotic and anti-inflammatory properties, exerting its therapeutic effects primarily by reducing fibroblast proliferation (7). Although its precise mechanism of action remains only partly understood, the molecule modulates the production of transforming growth factor-β1, a key profibrotic and pro-inflammatory cytokine. It also inhibits the differentiation of human lung fibroblasts into myofibroblasts, thereby limiting excessive collagen synthesis and extracellular matrix deposition (8–11). Clinical trials have demonstrated that *pirfenidone* is associated with improved pulmonary function, prolonged progression-free survival, enhanced exercise tolerance, and significant reduction in the overall progression of the disease (10–13).

Meanwhile, *nintedanib* employs a different mechanism of action. It is an indolinone derivative that functions as a small-molecule tyrosine kinase inhibitor, targeting multiple vascular endothelial growth factor receptors involved in fibrogenesis, thereby inhibiting fibroblast proliferation. Initially proposed for the treatment of non-small cell lung cancer, it has also been studied in IPF due to the overlap in pathogenic growth factor pathways (14). The molecule competitively binds to the intracellular adenosine triphosphate binding site of growth factor receptors, preventing their autophosphorylation and blocking downstream signaling cascades. *Nintedanib* directly blocks non-receptor tyrosine kinases, preventing fibroblast activation, thereby inhibiting fibroblast proliferation and migration, effectively attenuating angiogenesis in the lungs. It has been shown to significantly reduce the rate of decline in FVC compared to placebo (15) and to slow disease progression (16).

The fibrotic process in IPF is driven by abnormal fibroblast activation and excessive extracellular matrix deposition (17). Repeated alveolar epithelial injury triggers dysregulated immune responses, impaired DNA repair, and abnormal surfactant protein processing, leading to defective wound healing (18, 19). This cascade promotes progressive fibrosis, involving both innate and adaptive immunity, including Natural Killer (NK) cell activation (20). Their activity is regulated by a broad repertoire of activating and inhibitory receptors among which NKG2D is one of the most important (21). This receptor is expressed on the surface of NK cells and certain T cell subsets, and, upon activation, triggers the release of cytokines and cytotoxic mediators (20).

The main ligands of the NKG2D receptor are the non-classical MHC class I chain-related molecules A (MICA), encoded on chromosome 6 (22). Unlike classical *HLA* class I genes, MICA does not present antigenic peptides but functions as a polymorphic stress-induced ligand for NK cells. Normally restricted to the intestinal epithelium, MICA expression increases in response to tissue damage (23), promoting cytotoxic responses and modulating inflammation (24). The binding affinity to NKG2D is determined by a polymorphism at position 129: *MICA*-Met129 confers stronger receptor activation and enhanced NK cell cytotoxicity compared to *MICA*-Val129 (25, 26). This variant has been associated with several immune-mediated conditions, and prior studies have hypothesized a role in IPF susceptibility via modulation of NKG2D expression (27).

Based on this biological rationale, we focused on *MICA* as a functional immunogenetic candidate potentially involved in clinical heterogeneity and antifibrotic treatment response in IPF. The aim of this study was therefore to evaluate whether *MICA* polymorphisms, with particular attention to the functional Met129Val variant, were associated with disease progression, overall survival, and treatment-specific outcomes. We leveraged a well-phenotyped Sardinian cohort already genotyped for multiple MHC loci, an advantageous genetic isolate with relatively high homogeneity that is ideally suited to targeted immunogenetic association studies (28–31).

## 2 Materials and methods

### 2.1 Study cohorts

**Figure 1:**
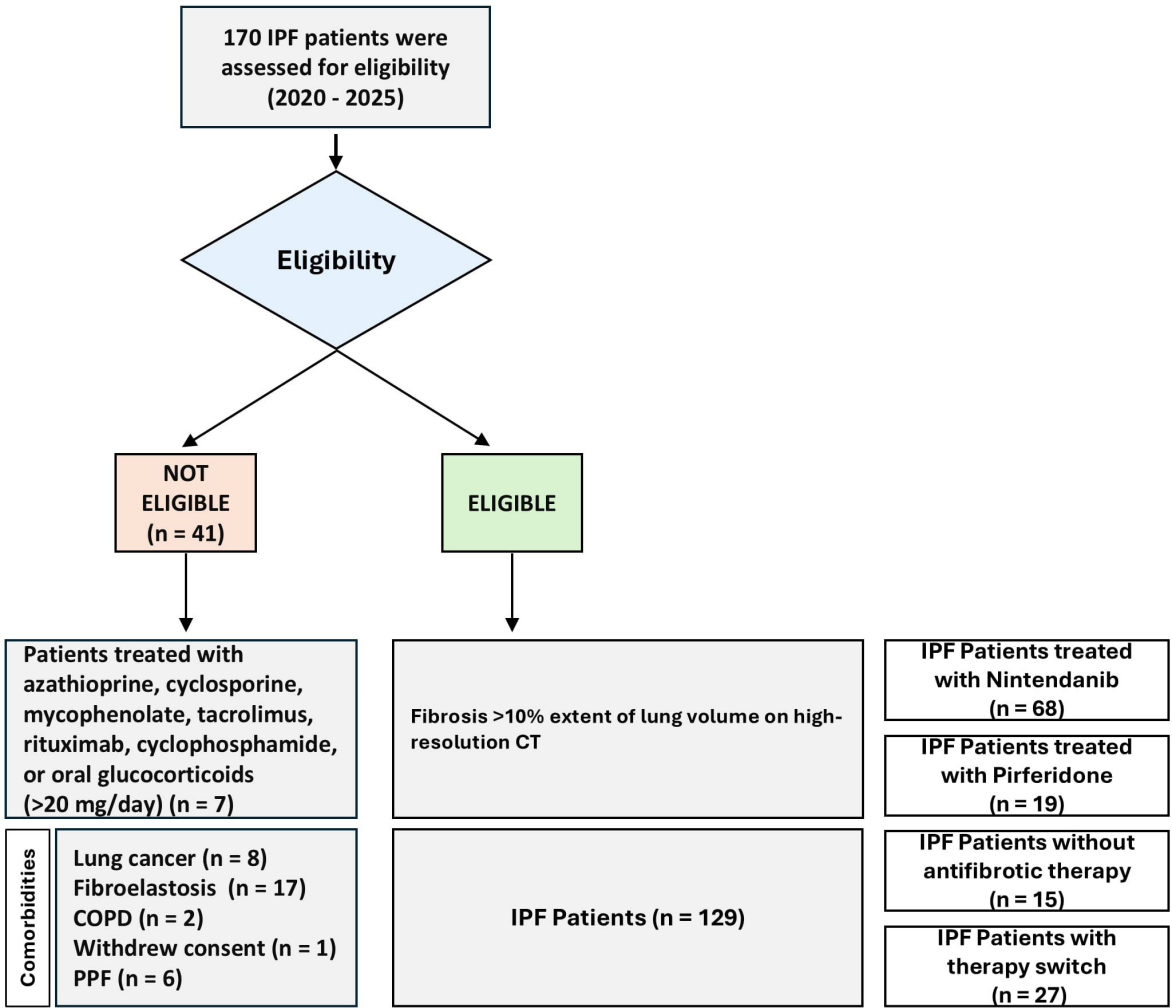
IPF Patients’ enrollment. A total of 170 IPF were followed at the Department of Pneumology of Binaghi Hospital (ASL, Cagliari) from May 2020 to May 2025. IPF was diagnosed according to the American Thoracic Society, European Respiratory Society, Japanese Respiratory Society, and Asociación Latinoamericano de Tórax guidelines and recommendations (32). COPD, Chronic obstructive pulmonary disease; ILD, Interstitial lung diseases; IPF, Idiopathic pulmonary fibrosis; PPF, Progressive pulmonary fibrosis.

Patient recruitment was conducted between May 2020 and May 2025. Adults (≥18 years) with a physician-confirmed diagnosis of fibrosing interstitial lung disease (ILD), consistent with international guidelines (ATS/ERS/JRS/ALAT/NICE 2017) (32), were considered eligible. Diagnosis was based on multidisciplinary evaluation, including clinical history, radiographic findings, HRCT (fibrosing lung involvement ≥10%), and pulmonary function tests: predicted FVC% and DLCO% (FVCp% and DLCOp%). These parameters were assessed at baseline and monitored during follow-up (12, 24, and 36 months). Demographic and clinical data collected at baseline included age, sex, smoking history, comorbidities, and extent of pulmonary impairment. In addition, mild or asymptomatic SARS-CoV-2 infection data were not available, therefore, not included in the study. Treatment decisions were made according to international guideline-based individualized approaches. To reduce treatment-related bias, 7 patients receiving immunosuppressants were excluded (**Figure 1**). An additional 34 patients were excluded due to pulmonary comorbidities such as lung cancer or obstructive disease. Of the 129 patients included, 68 received *nintedanib* (100–150 mg twice daily), and 19 were treated with *pirfenidone* according to clinical practice. A subgroup of 27 patients switched therapy due to drug intolerance or adverse effects: 16 patients switched from *nintedanib* to *pirfenidone* (59.3%), whereas 11 patients switched from *pirfenidone* to *nintedanib* (40.7%). A separate group did not receive antifibrotic treatment, mainly because of poor adherence, severe side effects, or advanced age.

### 2.2 DNA extraction and MICA genotyping

Peripheral blood was collected into 3 mL EDTA tubes. Samples were treated with a red blood cell lysis buffer (RCLB) for 10 minutes to eliminate erythrocytes, then centrifuged at 2500 × g for 10 minutes at 4 °C to separate plasma from the cellular fraction. The resulting peripheral blood mononuclear cell pellet was resuspended in 200 µL of phosphate-buffered saline and used for genomic DNA extraction with the QIAamp DNA Blood Mini Kit (Qiagen), following the manufacturer’s protocol. High-resolution genotyping of class I and class II *HLA* loci was performed using the AlloSeq Tx17 early pooling workflow, covering 17 loci (*HLA-A, -B, -C, -E, -F, -G, -H, -DRB1, -DRB3/4/5, -DQA1, -DQB1, -DPA1, -DPB1, -MICA, -MICB*). Libraries were prepared using the AlloSeq Tx17 kit (CareDx, USA), suitable for Illumina sequencing platforms. Paired-end sequencing (2 × 150 bp) was carried out on a MiSeq system (Illumina, USA). *HLA* typing was performed with AlloSeq Assign software (version 1.0.3, CareDx, USA), using reference allele information from the IPD-IMGT/HLA database (version 3.45.1.2).

### 2.3 Statistical methods

Descriptive statistics were computed for both clinical and genetic data of IPF patients. Continuous variables were calculated using means and standard deviations (SD) while categorical variables with proportions and odds ratios (ORs). Comparisons between groups were performed using Student’s t-test or Kruskal–Wallis test for continuous variables and Fisher’s exact test for categorical variables, as appropriate. All p-values were two-tailed, and statistical significance was set at p < 0.05. Allele and haplotype frequencies of *MICA* variants were obtained using custom scripts written in R (version 4.4.2; R Core Team, 2024. R: A language and environment for statistical computing. R Foundation for Statistical Computing, Vienna, Austria. URL: https://www.R-project.org/), which was also used to perform all statistical analyses. Kaplan–Meier survival curves were generated to evaluate overall survival (OS), defined as the time from diagnosis to death or last follow-up. Patients were stratified by antifibrotic treatment (*nintedanib*, *pirfenidone*, no therapy and switchers groups) and by *MICA*-129 Met/Val genotype. Survival analyses were performed using Kaplan–Meier estimates and Cox proportional hazards models. Multivariable Cox models were adjusted for age, sex, smoking status, medication and comorbidity burden, *nintedanib* dose and *HLA* extended haplotype. To evaluate whether the association between *MICA*-*129 Val/Val* status and OS differed according to antifibrotic treatment, a treatment-by-genotype interaction analysis was performed in patients receiving *nintedanib* or *pirfenidone* (**Supplementary Methods**). Survival analyses were adjusted using the Benjamini–Hochberg false discovery rate (FDR) method. Both raw and adjusted p-values were reported. The proportional hazards assumption was assessed using Schoenfeld residuals. Statistical analyses were performed in R version 4.4.2.

## 3 Results

### 3.1 Genetic and clinical stratification of IPF patients according to antifibrotic therapy

Across treatment groups, no therapy (n = 15), *nintedanib* (n = 68), *pirfenidone* (n = 19), and therapy switchers (n = 27) (**Supplementary Table 1**), we observed a marginally significant difference in sex distribution (p = 0.048), with a higher proportion of females in the no-therapy group versus those receiving antifibrotics. No significant differences were observed in smoking history or the use of long-term oxygen therapy (p = 0.167 and p = 0.580, respectively). Age at diagnosis varied by group, with untreated patients being older on average (76.7 ± 11.1 years) compared to those treated with *nintedanib* (69.4 ± 7.4 years), *pirfenidone* (67.4 ± 6.4 years), or those who switched therapy (69.0 ± 9.5 years) (*p* = 0.003).

Pulmonary function parameters at baseline were slightly lower in patients treated with *pirfenidone* (mean FVCp%: 74.6 ± 20.0; DLCOp%: 63.6 ± 17.0), while higher values were observed in the *nintedanib* group per FVCp% (FVCp%: 74.8 ± 19.6; DLCOp%: 58.7 ± 17.5). Better values were recorded among therapy switchers (FVCp%: 82.0 ± 18.6; DLCOp%: 65.0 ± 19.4) and untreated patients (FVCp%: 86.0 ± 12.9; DLCOp%: 74.4 ± 16.6). Although some differences in baseline lung function were noted, no statistically significant differences were found among groups. Pulmonary function was monitored over time, including at 12, 24, and 36 months. Across all time points, no significant intergroup differences emerged in the evolution of FVCp% and DLCO%p (**Supplementary Table 1**).

We compared *MICA* allele frequencies across the four treatment subgroups (**Supplementary Table 2**). In the overall cohort, the most common alleles were MICA*001:01 and MICA*002:01 (both 18.6%), followed by MICA*008:01 (11.6%), MICA*018:01 (9.7%), and MICA*004:01 (6.2%).

Modest between-group differences were observed. For instance, MICA*008:01 was more frequent in the *nintedanib* group (15.4%) than in the *pirfenidone* (5.3%) and no-therapy (6.7%) groups, whereas MICA*004:01 was comparatively enriched among untreated patients (13.3%). However, none of these differences reached statistical significance (p > 0.05 for all comparisons).

The distribution of rare alleles (frequency < 5%) likewise did not differ across treatment groups (p > 0.05). We also examined variation at codon 129 of the *MICA* gene, which results in a methionine (Met) to valine (Val) substitution, a polymorphism known to influence the binding affinity of MICA to the NKG2D receptor (**Supplementary Table 3**). Neither allele nor genotype frequencies varied by treatment (p = 0.620), and all three genotypes (Met/Met, Met/Val, Val/Val) were represented in every subgroup.

### 3.2 Overall survival analysis

**Figure 2:**
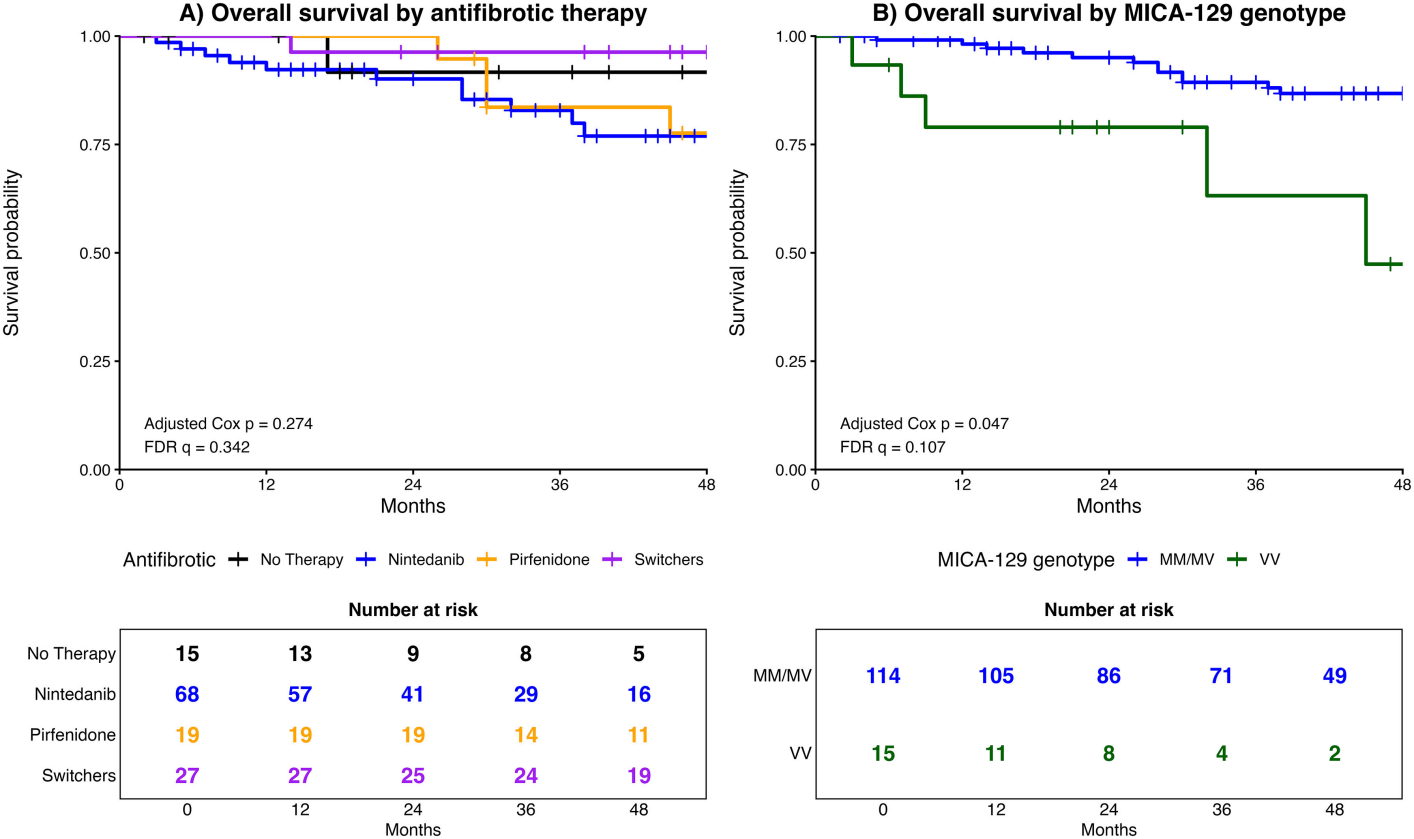
Overall survival stratified by antifibrotic therapy and MICA-129 Met/Val genotype in IPF patients. **(A)** Kaplan–Meier curves showing overall survival at 48 months in patients grouped by antifibrotic treatment (No Therapy, Nintedanib, Pirfenidone, and Switchers). **(B)** Kaplan–Meier survival curves according to MICA-129 genotype (MM/MV, VV).

The Kaplan–Meier survival curve (**Supplementary Figure 1**) illustrates the OS of the 129 IPF patients included in the study. At 48 months, the estimated OS was 81.5% (51/129 patients at risk; 95% CI: 73.9–89.9%). The median follow-up duration among surviving patients was 41.5 months. To explore potential differences in survival according to treatment, patients were stratified by antifibrotic therapy (**Figure 2A**). At 48 months, the estimated OS was 73.3% for untreated patients (95% CI: 45.8–100%), 59.3% for those treated with *nintedanib* (95% CI: 38.8–77.6%), 73.3% for patients treated with *pirfenidone* (95% CI: 44.9–92.2%), and 71.0% for therapy switchers (95% CI: 53.7–93.8%).

Survival curves were generated to evaluate OS according to antifibrotic treatment and *MICA-129* status under a recessive model, comparing *Val/Val* carriers with *Met/Met–Met/Val* patients. Before interpreting treatment-stratified survival analyses, we note that genotype-defined subgroups were small, particularly for *Val/Val* carriers representing 10/68 nintedanib-treated patients and 3/19 *pirfenidone*-treated patients.

In the overall cohort, OS did not differ significantly across treatment groups in adjusted Cox analysis (**Figure 2A**; adjusted Cox p = 0.290, FDR q = 0.363, χ² = 3.70). When stratified by MICA-129 status, *Val/Val* carriers showed poorer OS than *Met/Met–Met/Val* patients in the overall cohort, although this association did not remain significant after correction for multiple testing (**Figure 2B**; adjusted Cox p = 0.044, FDR q = 0.089, χ² = 4.05); deaths occurred in 12/114 (10.5%) *Met/Met–Met/Val* patients and 5/15 (33.3%) *Val/Val* carriers. The estimated OS was 80.3% (95% CI: 68.2–89.4%) for *Met/Met–Met/Val* patients and 47.4% (95% CI: 3.7–71.0%) for *Val/Val* patients.

**Figure 3:**
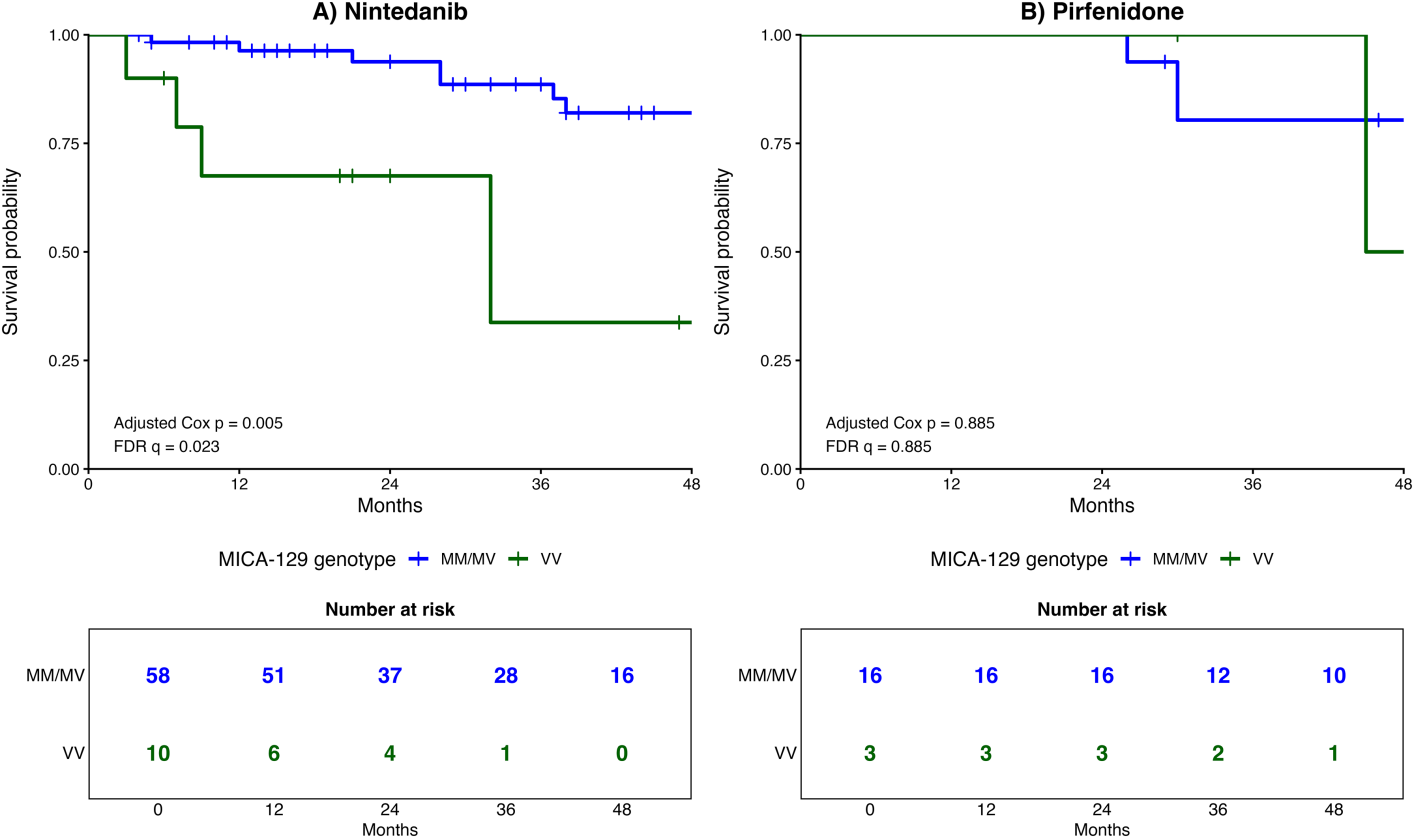
Overall survival stratified by MICA-129 Met/Val genotype in IPF patients treated with Nintedanib or Pirfenidone. **(A)** Kaplan–Meier survival curves in patients treated with Nintedanib stratified by MICA-129 genotype (MM/MV, VV). **(B)** Kaplan–Meier survival curves in patients treated with Pirfenidone stratified by MICA-129 genotype.

We next examined OS within the *nintedanib* and *pirfenidone* subgroups (**Figures 3A, B**). Among patients treated with *nintedanib*, *Val/Val* carriers showed markedly reduced OS compared with for *Met/Met–Met/Val* patients [33.8% (95% CI: 7.9–100%) vs 69.6% (95% CI: 47.1–86.8%)] (**Figure 3A**; adjusted Cox p = 0.005, FDR q = 0.023). Deaths within 48 months occurred in 4/10 (40.0%) *Val/Val* carriers and 7/58 (12.1%) *Met/Met–Met/Val* patients. In contrast, no significant difference was observed among patients treated with pirfenidone (**Figure 3B**; adjusted Cox p = 0.690; 1/3 [33.3%] *Val/Val* vs 3/16 [18.8%] *Met/Met–Met/Val* deaths; OS 50.0% [95% CI: 1.3–98.7%] and 76.9% [95% CI: 46.2–95.0%], respectively). We then performed an additional multivariable Cox regression analysis restricted to *nintedanib*-treated patients. In this model, MICA-129 genotype was analyzed under a recessive model by comparing *Val/Val* carriers with *Met/Met–Met/Val* patients, and the analysis was adjusted for age, sex, smoking status, *nintedanib* dose, extended *HLA* haplotype, medication burden, and comorbidity burden. Patients carrying *Val/Val* showed a significantly increased mortality risk compared with *Met/Met–Met/Val* patients (HR = 7.59, 95% CI: 1.83–31.53; p = 0.01; **Supplementary Figure 3**). Sensitivity analyses showed consistent associations across additional models accounting for concomitant medications and comorbidities (p < 0.05, **Supplementary Table 5**).

To further address potential baseline imbalance between genotype groups, we performed a propensity score–weighted sensitivity analysis among *nintedanib*-treated patients. The propensity score was estimated using the same set of covariates. Stabilized inverse probability weighting improved covariate balance, with five of six covariates achieving an absolute standardized mean difference below 0.10; medication burden showed only slight residual imbalance (**Supplementary Figure 4**). In the IPTW-weighted Cox model with robust standard errors, MICA-129 Val/Val remained associated with poorer OS compared with MM/MV patients (HR = 5.22, 95% CI: 1.57–17.39; p = 0.0071). To formally assess whether the association between MICA-129 *Val/Val* genotype and OS differed according to antifibrotic treatment, we fitted a multivariable Cox proportional hazards model restricted to patients treated with *nintedanib* or *pirfenidone* and including a treatment-*MICA-129 Val/Val* interaction. The treatment-genotype interaction did not reach statistical significance (HR = 4.02, 95% CI: 0.33–49.54; p = 0.277; likelihood-ratio test p = 0.248).

Finally, we analyzed adjusted trajectories of FVC% predicted and DLCO% predicted over 36 months. In *nintedanib*-treated patients, mixed-effects models showed no significant genotype-by-time interaction for changes in FVC% predicted (p = 0.697) or DLCO% predicted (p = 0.696). *Val/Val* carriers displayed a less favorable adjusted FVC trajectory, but this pattern did not reach statistical significance and was not paralleled by a consistent DLCO% decline (**Supplementary Figure 2**).

## 4 Discussion

In this study, we investigated the role of *MICA* polymorphisms in a genetically homogeneous cohort of Sardinian patients with IPF, focusing on the *MICA*-*129 Met/Val* variant and its potential association with clinical outcomes.

In the absence of head-to-head randomized trials, antifibrotic therapy was individualized: *pirfenidone* at higher bleeding risk (e.g., on full-dose anticoagulation or dual antiplatelet therapy) given its more favorable safety profile in this context (33); this strategy reflects guidance and trial data indicating that *nintedanib* can modestly increase bleeding events, typically minor (epistaxis, bruising) (34).

Conversely, *nintedanib* was favoured for patients with dermatologic comorbidities or occupational sun exposure to avoid *pirfenidone-*related photosensitivity (35). This pragmatic stratification aimed to maximise safety and adherence without sacrificing efficacy, and, consistent with prior work, overall survival was comparable between *nintedanib* and *pirfenidone* (**Figure 2A**) (36, 37).

Although *MICA* allele and genotype frequencies did not differ significantly across treatment groups (**Supplementary Table 2**), our data suggest that the *MICA*-129 Val/Val genotype is linked to poorer prognosis, particularly among patients treated with *nintedanib*. Kaplan–Meier curves show that Val/Val carriers receiving *nintedanib* had a significantly reduced 48-month overall survival compared to Met/Met and Met/Val patients (p-value = 0.005; FDR q = 0.023; **Figure 3A**). This pattern is not seen in those treated with *pirfenidone* (**Figure 3B**; p-value> 0.05). The adverse survival signal in *nintedanib*-treated *Val/Val* carriers was supported by a multivariable Cox model in patients receiving nintedanib and adjusted for age, sex, smoking status, *nintedanib* dose, extended *HLA* haplotype, medication burden, and comorbidity burden (HR = 7.59 (1.83–31.53); p = 0.01). The effect size was large, although the confidence interval was wide, reflecting the limited number of *Val/Val* carriers and mortality events (**Supplementary Figure 3**). Importantly, this association remained evident after accounting for clinical variables, suggesting that the observed effect was not fully explained by measured baseline differences. To further address potential imbalance between genotype groups, we performed a propensity score–weighted sensitivity analysis within the *nintedanib*-treated subgroup. Stabilized inverse probability weighting improved covariate balance across most baseline variables. In the weighted Cox model, *MICA-129 Val/Val* remained significantly associated with poorer overall survival (HR = 5.22, 95% CI: 1.57–17.39, p = 0.0071). This analysis strengthens the internal consistency of the association between *Val/Val* status and adverse outcome among *nintedanib*-treated patients (**Supplementary Figure 4**).

Although the Val/Val-associated survival disadvantage was most pronounced among *nintedanib*-treated patients, the formal treatment-by-genotype interaction did not reach statistical significance (likelihood-ratio test p = 0.248). This result should be interpreted considering the limited number of *Val/Val* carriers and mortality events within treatment groups, which reduced the statistical power to detect interaction effects.

Finally, adjusted longitudinal analyses of FVC% predicted and DLCO% predicted did not show significant genotype-by-time interactions over 36 months. Although Val/Val carriers showed a numerically less favorable FVC trajectory, this finding remained exploratory and was not supported by a consistent DLCO % pattern.

Previous clinical trials, including the two replicate 52-week randomized, double-blind phase III studies INPULSIS-1 and INPULSIS-2, demonstrated that *nintedanib* effectively slows disease progression by reducing the decline in FVC compared to placebo (6). Our data highlight, however, that over the long term, mortality may be influenced by specific genetic background.

In the same Sardinian IPF cohort, earlier work identified an *HLA* extended haplotype (HLA-A*30:02, -B*18:01, -C*05:01, -DQA1*05:01, -DQB1*02:01, -DRB1*03:01) and the DRB1*04:05 allele as independent positive prognostic factors for survival (38). In the multivariate analysis (**Supplementary Table 4**), the *MICA*-129 Val/Val genotype remained significantly associated with poorer survival outcomes, although the effect size was attenuated after adjustment for the extended *HLA* haplotype.

Growing evidence indicates that non-classical *HLA* genes have an immunogenetic role in shaping treatment response. Indeed, a study has demonstrated that the Val/Val (*rs1051792 GG*) variant is nominally associated with poorer outcomes in patients treated with anti-TNF therapies (39). In Rheumatoid arthritis, patients carrying the GG genotype showed a significantly reduced response after 3 months of anti-TNF treatment compared to those with GA or AA genotypes (39). That said, the effect is context-dependent: unlike our findings, and those in Rheumatoid arthritis (39), a recent study in axial spondylarthritis treated with TNF inhibitors reported that the A allele (Met129) was more frequent in patients than controls and was associated with worse treatment response (40).

The biological plausibility of our findings is supported by the functional role of the *MICA-129 Met/Val* variant in modulating MICA–NKG2D receptor interaction. The Met129 isoform binds NKG2D with higher affinity, triggering stronger NKG2D-dependent immune response (25). This leads to enhanced NK cell cytotoxicity, increased IFN-γ production, and more efficient CD8+ T cell co-stimulation (39). However, these effects are tightly regulated through a feedback mechanism: high expression levels of MICA-129Met induce NKG2D downregulation, thereby preventing sustained immune activation (25). By contrast, the Val129 isoform exhibits lower NKG2D affinity, greater cell-surface stability and is more readily shed as soluble MICA (41). Notably, elevated expression of the *MICA-129 Val* appears less effective at inducing NKG2D downregulation, potentially leading to sustained activation of NK and T cells and contributing to a dysregulated immune response (39).

This dysregulated MICA–NKG2D interaction may blunt the effectiveness of immunomodulatory treatments, including *nintedanib*. Although *nintedanib* is best known as a triple tyrosine kinase inhibitor targeting pro-fibrotic signaling in IPF, increasing data highlight additional anti-inflammatory actions (42). Preclinical studies have demonstrated that *nintedanib* reduces cytokine expression (including TNF-α, IL-1β, and IL-6) (43), inhibits macrophage infiltration, and mitigates tissue remodeling and inflammation across Asthma, Peritoneal fibrosis, and Rheumatoid arthritis models (44, 45). Moreover, it has shown protective effects against TNF-α–induced oxidative stress and extracellular matrix degradation in chondrocyte models (46).

Taken together, these findings suggest that in individuals with the *MICA-129 Val/Val* genotype, the persistent activation of the NKG2D pathway may counteract the anti-inflammatory effects of *nintedanib*, thereby limiting its therapeutic benefit. This interaction between genetic background and drug response might explain the observed reduced survival in this subgroup, despite standard antifibrotic therapy. However, because longitudinal FVC% predicted and DLCO% predicted trajectories did not show significant genotype-by-time interactions, the present data do not allow us to conclude that Val/Val directly reduces the functional efficacy of *nintedanib*. The main strength of this study is the integration of high-resolution immunogenetic data with longitudinal clinical outcomes in a genetically homogeneous cohort. The consistency of the *Val/Val* survival association across Kaplan–Meier, multivariable Cox, and IPTW-weighted sensitivity analyses supports the robustness of the observed signal within the *nintedanib*-treated subgroup. While encouraging, these findings must be interpreted with caution. The retrospective and observational design precludes definitive conclusions regarding direct causality, and the relatively small sample size, especially within treatment-by-genotype strata, may limit the generalizability of the findings. Wide confidence intervals around the Cox estimates reflects this limited precision. In addition, the formal treatment-by-genotype interaction was not statistically significant, preventing definitive conclusions about a treatment-specific predictive effect. Given the small size of the *pirfenidone* subgroup and the limited number of *Val/Val* carriers, the lack of significance in this group should not be interpreted as evidence of no effect. Larger, balanced cohorts are needed to determine whether the observed association is treatment-specific or reflects a broader prognostic effect. We therefore position this work as a discovery study that generates a testable signal. Independent replication in larger, multi-center cohorts and, ideally, validation within randomized controlled trials is needed to confirm the predictive value of *MICA* genotyping. Embedding immunogenetic profiling in future trial designs could elucidate determinants of antifibrotic response and accelerate truly personalized management of IPF.

## Data Availability

The data presented in this study are deposited in the repository, DOI: [https://doi.org/10.5281/zenodo.21217421]. Further inquiries can be directed to the corresponding authors.
