# Supplementary material for: MICA-129Met/Val as a therapeutic compass in idiopathic pulmonary fibrosis: prognosis and antifibrotic benefit
Source: Front Immunol. 2026 Jul 20;17:1722213. doi: 10.3389/fimmu.2026.1722213 (PMC13429406; doi:10.3389/fimmu.2026.1722213)
Supplement: Supplementary file 1 [file DataSheet1.pdf]

	Total Patients (n = 129)	No Therapy (n = 15)	*Nintedanib* (n = 68)	*Pirfenidone* (n = 19)	Therapy Switchers (n =27)	p-value
Age at diagnosis	69.9 ± 8.5	76.7 ± 11.1	69.4 ± 7.4	67.4 ± 6.4	69.0 ± 9.5	**0.003**
Age < 65 yo	25 (19.4%)	1 (6.7%)	12 (17.6%)	6 (31.6%)	6 (22.2%)	0.318
Sex						**0.048**
Male	103 (79.8%)	14 (93.3%)	58 (85.3%)	14 (73.7%)	17 (63.0%)	
Female	26 (20.2%)	1 (6.7%)	10 (14.7%)	5 (26.3%)	10 (37.0%)	
Smoking						0.167
No	10 (7.8%)	0 (0.0%)	10 (14.7%)	0 (0.0%)	0 (0.0%)	
Yes	32 (24.8%)	5 (33.3%)	16 (23.5%)	5 (26.3%)	6 (22.2%)	
Former Smokers	87 (67.4%)	10 (66.7%)	42 (61.8%)	14 (73.7%)	21 (77.8%)	
Oxygen Therapy						0.580
No	44 (34.1%)	3 (20.0%)	23 (33.8%)	8 (42.1%)	10 (37.0%)	
Yes	85 (65.9%)	12 (80.0%)	45 (66.2%)	11 (57.9%)	17 (63.0%)	
Pulmonary function parameters						
FVCp (%) 0 Month	77.1 ± 19.2	86.0 ± 12.9	74.8 ± 19.6	74.6 ± 20.0	82.0 ± 18.6	0.221
FVCp (%) 12 Months	77.3 ± 19.5	89.5 ± 10.6	77.6 ± 20.7	70.9 ± 15.6	79.9 ± 20.3	0.482
FVCp (%) 24 Months	72.8 ± 20.2	86.5 ± 2.1	74.9 ± 20.9	65.7 ± 20.8	74.0 ± 18.9	0.170
FVCp (%) 36 Months	71.7 ± 17.3	84.0 ± 14.5	72.7 ± 19.1	66.7 ± 17.3	73.4 ± 15.8	0.610
DLCOp (%) 0 Month	61.9 ± 18.0	74.4 ± 16.6	58.7 ± 17.5	63.6 ± 17.0	65.0 ± 19.4	0.160
DLCOp (%) 12 Months	61.4 ± 17.9	68.0 ± 15.2	59.9 ± 17.6	62.5 ± 19.3	63.1 ± 19.0	0.852
DLCOp (%) 24 Months	58.7 ± 17.8	76.0 ± 12.5	55.6 ± 18.1	61.8 ± 17.3	59.1 ± 18.3	0.271
DLCOp (%) 36 Months	53.6 ± 17.6	77.0 ± 14.3	50.3 ± 18.3	57.5 ± 15.9	53.5 ± 17.7	0.337

Table S1. *Clinical and Demographic Characteristics of IPF Patients by Treatment Group.*
Baseline and longitudinal characteristics of idiopathic pulmonary fibrosis (IPF) patients (n = 129), grouped by treatment: No Therapy (n = 15), Nintedanib (n = 68), Pirfenidone (n = 19), and Therapy Switchers (n = 27). Data are expressed as mean ± standard deviation or number (percentage), with corresponding p-values for group comparisons.
**p-value: P-values were computed to assess differences between treatment groups (No Therapy, Nintedanib, Pirfenidone, and Therapy Switchers) in both continuous and categorical variables.*
Note: p-values in bold indicate results statistically significant (p < 0.05).

MICA **Allele**	**Total (2N)**	**Allele Frequency (%)**	**No Therapy (2N)**	**Allele Frequency (%)**	***Nintedanib*** **(2N)**	**Allele Frequency (%)**	***Pirfenidone*** **(2N)**	**Allele Frequency (%)**	**Therapy Switchers (2N)**	**Allele Frequency (%)**	**p-value**
001:01	48	18.6	9	30	25	18.4	5	13.2	9	16.7	0.3266
002:01	48	18.6	5	16.7	27	19.9	6	15.8	10	18.5	0.9374
008:01	30	11.6	2	6.7	21	15.4	2	5.3	5	9.3	0.2813
018:01	25	9.7	1	3.3	15	11	3	7.9	6	11.1	0.6633
004:01	16	6.2	4	13.3	6	4.4	2	5.3	4	7.4	0.2797
009:01	15	5.8	1	3.3	7	5.1	4	10.5	3	5.6	0.6377
016:01	14	5.4	1	3.3	10	7.4	2	5.3	1	1.9	0.5595
012:01	12	4.7	2	6.7	5	3.7	4	10.5	1	1.9	0.1785
008:04	11	4.3	1	3.3	6	4.4	2	5.3	2	3.7	0.965
027:01	8	3.1	0	0	2	1.5	3	7.9	3	5.6	0.0792
011:01	7	2.7	1	3.3	3	2.2	1	2.6	2	3.7	0.8332
007:01	6	2.3	2	6.7	2	1.5	2	5.3	0	0	0.0834
017:01	4	1.6	0	0	1	0.7	2	5.3	1	1.9	0.1737
009:02	4	1.6	0	0	1	0.7	0	0	3	5.6	0.1229
011:01Q	3	1.2	0	0	1	0.7	0	0	2	3.7	0.2827
029:01	2	0.8	1	3.3	1	0.7	0	0	0	0	0.3457
019:01	2	0.8	0	0	1	0.7	0	0	1	1.9	0.7231
006:01	1	0.4	0	0	1	0.7	0	0	0	0	1
068:01	1	0.4	0	0	0	0	0	0	1	1.9	0.4729
010:01	1	0.4	0	0	1	0.7	0	0	0	0	1

Table S2. *Distribution of MICA Alleles by Treatment Group. The table shows the frequency (n, %) of each MICA allele observed in the total cohort and stratified by treatment group. Comparisons between groups were assessed using Fisher's exact test.*

MICA-129	Total (N)	No Therapy	*Nintedanib*	*Pirfenidone*	Therapy Switchers	p-value
MM	42 (32.6%)	6 (40.0%)	22 (32.4%)	7 (36.8%)	7 (25.9%)	0.782
MV	72 (55.8%)	9 (60.0%)	36 (52.9%)	9 (47.4%)	18 (66.7%)	0.537
VV	15 (11.6%)	0 (0.0%)	10 (14.7%)	3 (15.8%)	2 (7.4%)	0.396
M	156 (60.5 %)	21 (70.0 %)	80 (58.8 %)	23 (60.5 %)	32 (59.3 %)	0.723*
V	102 (39.5 %)	9 (30.0 %)	56 (41.2 %)	15 (39.5 %)	22 (40.7 %)	

Table S3. *Frequency of MICA-129 genotypes (Met/Met, Met/Val, Val/Val) and allelic (Met or Val) among IPF patients by treatment group. Statistical comparisons were performed using Fisher's exact test; *degree of freedom= 3.*

**Variable**	**HR (95% CI)**	**p-value**	**Model**
MICA-129 MV	0.74 (0.23–2.35)	0.608	A
MICA-129 VV	4.11 (1.00–16.83)	**0.049**	A
MICA-129 MV	1.06 (0.32–3.51)	0.925	B
MICA-129 VV	5.28 (1.28–21.69)	**0.021**	B

Table S4*. Multivariate Cox proportional hazards models for overall survival in IPF patients treated with nintedanib. Hazard ratios (HR) with 95% confidence intervals (CI) and p-values for MICA-129 genotypes and immunogenetic covariates, from two separate models. Model A is adjusted for the extended HLA haplotype (HLA-A*30:02,-B*18:01,-C*05:01,-DQA1*05:01,-DQB1*02:01,-DRB1*03:01), and Model B is adjusted for the HLA-DRB1*04:05 allele. The MM genotype serves as the reference category for MICA-129.*

Additional adjustment	HR VV vs MM/MV (95% CI)	p-value
Age, sex, smoking, nintedanib dose	6.32 (1.78–22.45)	0.004
Hypercholesterolemia	5.89 (1.65–21.00)	0.006
Pulmonary hypertension	7.07 (1.89–26.51)	0.004
History of malignancy	6.76 (1.80–25.30)	0.005
Diabetes mellitus	6.74 (1.77–25.69)	0.005
Antihypertensive therapy	5.86 (1.63–21.05)	0.007
Gastroprotective / anti-reflux therapy	7.96 (2.05–30.98)	0.003
Lipid-lowering therapy	7.51 (1.86–30.31)	0.005
Anticoagulant therapy	6.31 (1.79–22.29)	0.004
Antidiabetic therapy	6.06 (1.64–22.38)	0.007

Table S5. Sensitivity Cox regression analyses for overall survival in nintedanib-treated patients. *Hazard ratios were estimated in nintedanib-treated patients using MICA-129 Met/Met–Met/Val as the reference group. The base Cox model included age, sex, smoking status, and nintedanib dose. Additional Cox models included each medication or comorbidity variable individually.*

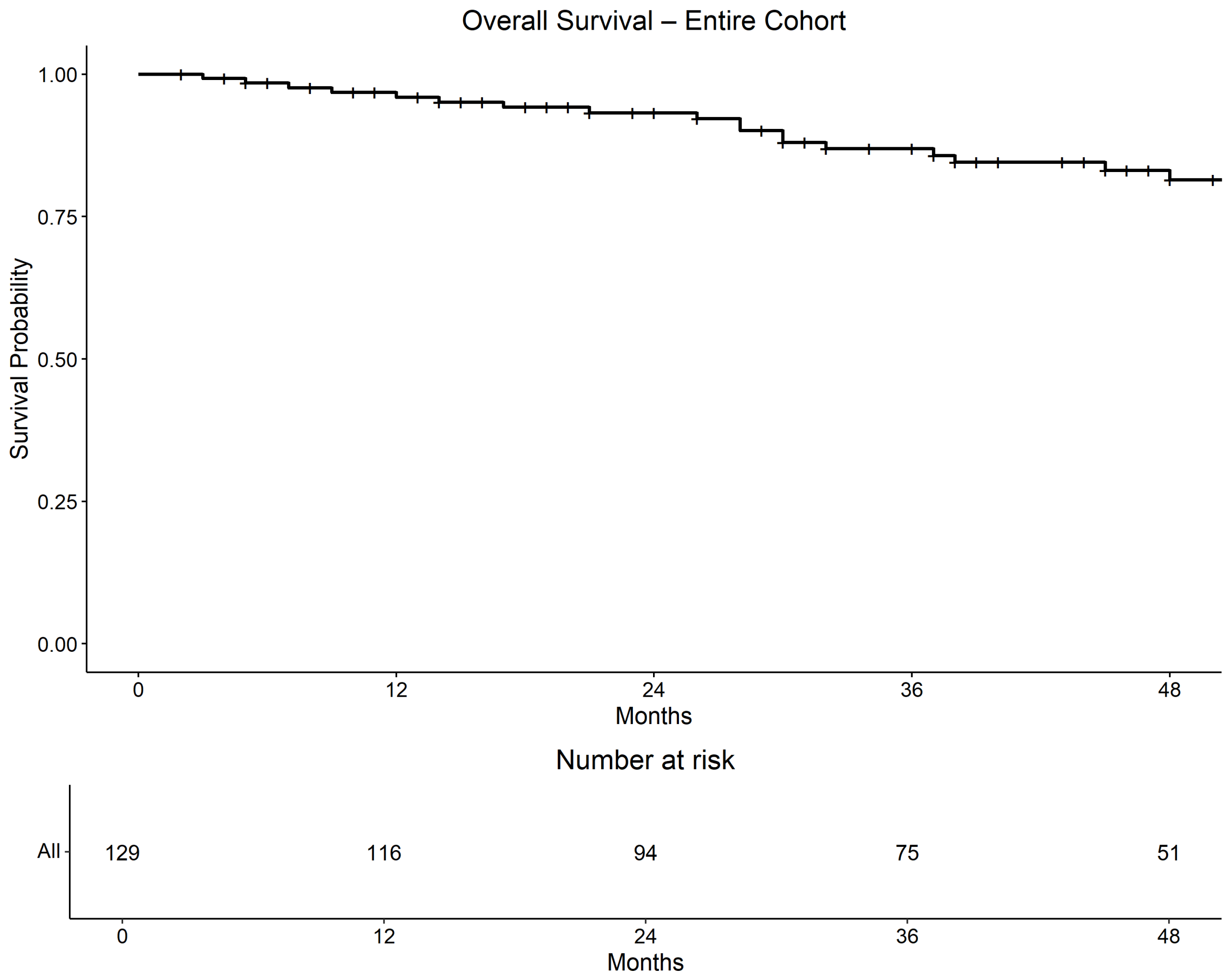

Figure S1. Kaplan–Meier survival curve for the IPF cohort over 48 months period. *The plot shows overall survival from the time of diagnosis up to 48 months in the full cohort (n = 129). Survival probability is indicated on the y-axis, and the number of patients at risk at each time is shown below the x-axis.*

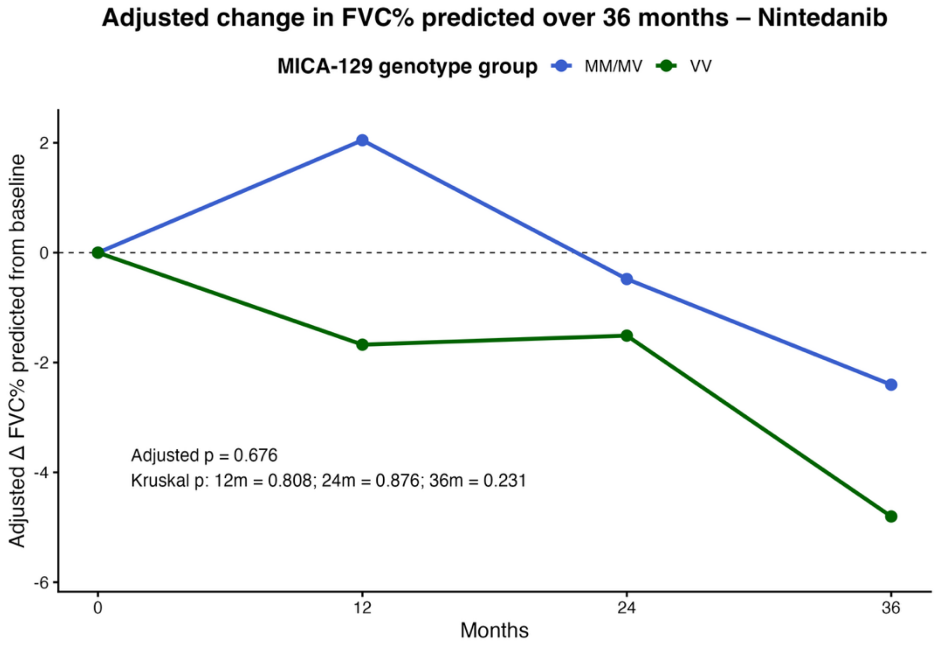

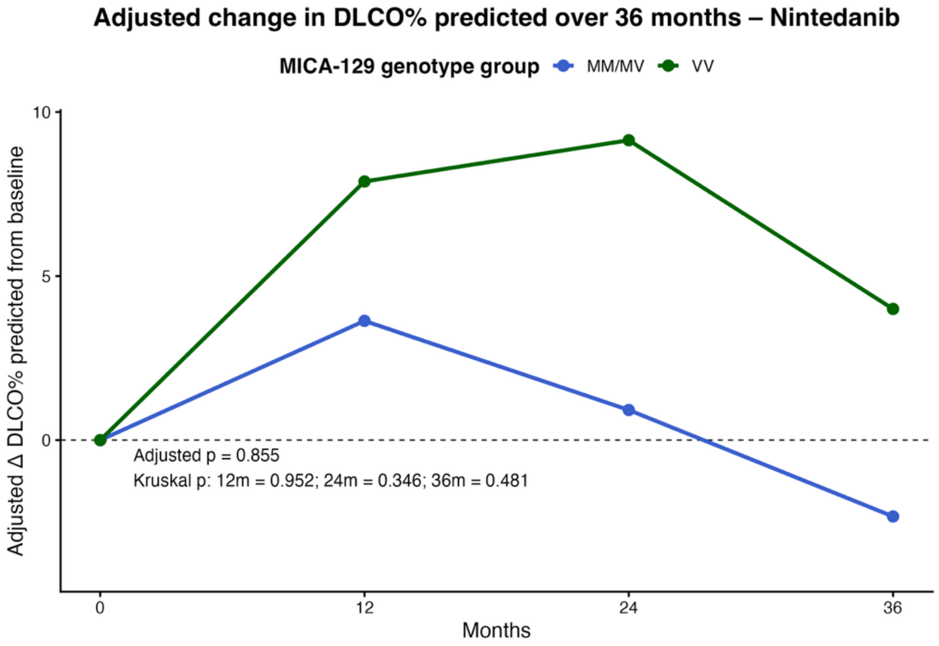

Figure S2. *Adjusted longitudinal changes in pulmonary function among nintedanib-treated patients according to MICA-129 genotype. Curves show adjusted mean changes from baseline in FVC% predicted and DLCO% predicted over 36 months, estimated using linear mixed-effects models adjusted for age at diagnosis, sex, smoking status, medication burden, and comorbidity burden. Shaded areas indicate 95% confidence intervals. The p-value shown in each panel refers to the genotype-by-time interaction and the Kruskal p-value over different time point.*

Variable		N	Hazard ratio		p
**Genotype**	MM/MV	58		Reference	
	VV	10		7.59 (1.83, 31.53)	0.01
**Age**		68		0.98 (0.90, 1.07)	0.68
**Sex**	F	10		Reference	
	M	58		0.58 (0.15, 2.31)	0.44
**Smoke**	Never	16		Reference	
	Ever	52		0.75 (0.20, 2.76)	0.66
**Nintedanib**	2 x 150 mg	27		Reference	
	2 x 100 mg	41		2.16 (0.65, 7.24)	0.21
**Haplotype**	Absent	58		Reference	
	Present	10		0.35 (0.04, 2.96)	0.33
**Medication burden**		68		0.60 (0.37, 1.00)	0.05
**Comorbidity burden**		68		1.30 (0.90, 1.89)	0.16

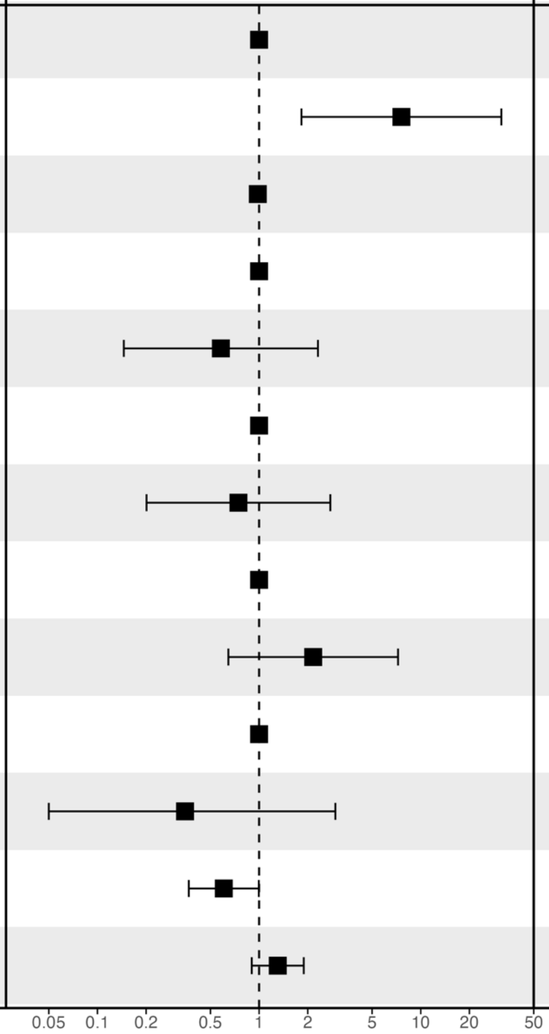

Figure S3. Forest plot of the multivariable Cox model in patients treated with nintedanib.

*The plot displays hazard ratios (HRs) and 95% confidence intervals for overall survival in nintedanib-treated patients. MICA-129 genotype was analysed under a recessive model, comparing Val/Val carriers with Met/Met–Met/Val patients, with the Met/Met–Met/Val group serving as the reference category. The model was adjusted for age, sex, smoking history coded as never versus ever smoker, nintedanib dose, extended HLA haplotype, medication burden, and comorbidity burden. Medication burden and comorbidity burden were calculated as the total number of recorded concomitant medication categories and comorbidities, respectively. HLA Haplotype refers to the presence of the previously described Sardinian HLA haplotype HLA-A*30:02, HLA-B*18:01, HLA-C*05:01, HLA-DQA1*05:01, HLA-DQB1*02:01, and HLA-DRB1*03:01.*

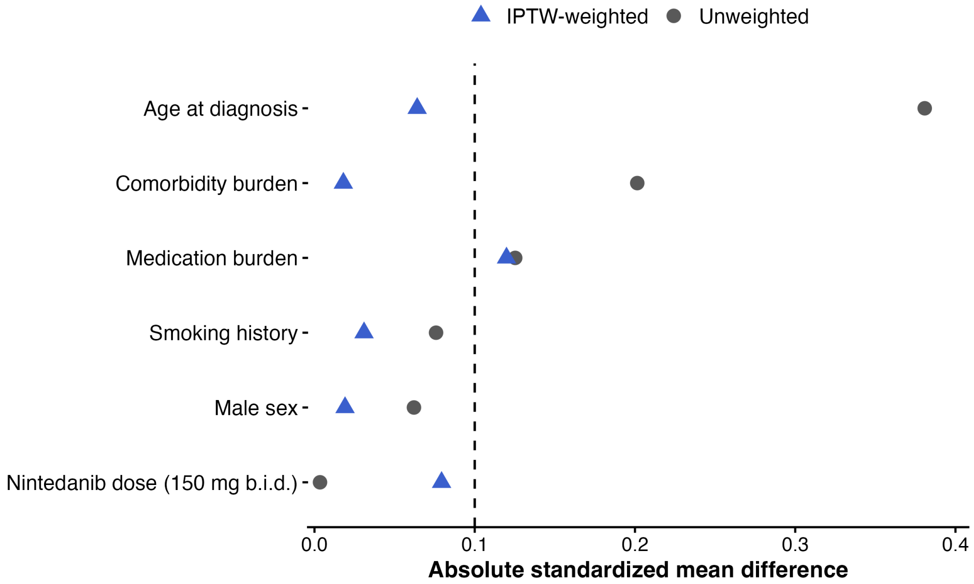

Figure S4. *Covariate balance before and after propensity score weighting.*

Absolute standardized mean differences are shown for baseline covariates included in the propensity score model comparing MICA-129 VV carriers with MM/MV patients among nintedanib-treated subjects. Grey circles represent the unweighted sample, and blue triangles represent the IPTW-weighted sample. The dashed vertical line indicates the prespecified balance threshold of 0.10. Covariates included age at diagnosis, sex, smoking history, nintedanib dose, medication burden, and comorbidity burden. IPTW, inverse probability of treatment weighting.

Supplementary Methods

Survival analyses and multiple-testing correction

Overall survival (OS) was defined as the time from diagnosis to death or last available follow-up. Kaplan–Meier curves were generated to visualize survival probabilities. Patients were stratified by antifibrotic treatment group and by MICA-129 genotype under a recessive model, comparing Val/Val carriers with Met/Met–Met/Val patients. Observed numbers at risk were calculated at 0, 12, 24, 36, and 48 months and displayed below each survival panel. To account for potential confounding, adjusted Cox proportional hazards models were fitted using predefined covariates. Benjamini–Hochberg false discovery rate correction was applied across the predefined Cox survival analyses, and both raw p-values and FDR-adjusted q-values were reported. The proportional hazards assumption was assessed using Schoenfeld residuals.

Multivariable Cox analysis in nintedanib-treated patients

To evaluate the association between MICA-129 genotype and overall survival among patients receiving nintedanib, we performed a multivariable Cox proportional hazards regression analysis restricted to the nintedanib-treated subgroup. Overall survival was defined as the time from diagnosis to death or last follow-up, with living patients censored at the date of last available follow-up. MICA-129 genotype was analyzed under a recessive genetic model by comparing Val/Val carriers with Met/Met–Met/Val patients. The Met/Met–Met/Val group was used as the reference category. The model was adjusted for age at diagnosis, sex, smoking history, nintedanib dose, extended HLA haplotype (*HLA-A*30:02, HLA-B*18:01, HLA-C*05:01, HLA-DQA1*05:01, HLA-DQB1*02:01, and HLA-DRB1*03:01*), medication burden, and comorbidity burden. Smoking history was recoded as never versus ever smoker, with current and former smokers grouped together. Nintedanib dose was modeled as 2 × 100 mg versus 2 × 150 mg, with 2 × 150 mg used as the reference category. Medication burden was calculated as the total number of recorded concomitant medication categories for each patient, including psychotropic medications, gastroprotective/anti-reflux therapy, antihypertensive therapy, antiplatelet therapy, anticoagulant therapy, lipid-lowering therapy, and antidiabetic therapy. Comorbidity burden was calculated as the total number of recorded comorbidities, including pulmonary hypertension, cardiac disease, systemic hypertension, hypercholesterolemia, diabetes mellitus, gastroesophageal reflux disease, obesity, chronic kidney disease, and history of malignancy. Hazard ratios (HRs), 95% confidence intervals (CIs), and p-values were estimated from the Cox model. Analyses were performed using complete cases for all variables included in the model. Statistical analyses were conducted in R using the survival package, and results were visualized as a forest plot.

Propensity score–weighted sensitivity analysis

Potential baseline imbalance between MICA-129 genotype groups were assessed using a propensity score–weighted sensitivity analysis among patients treated with nintedanib. MICA-129 genotype was analyzed under a recessive model, comparing VV carriers with MM/MV patients. The propensity score, defined as the probability of carrying the VV genotype conditional on baseline covariates, was estimated using logistic regression. Covariates included age at diagnosis, sex,

smoking history, nintedanib dose, medication burden, and comorbidity burden. Smoking history was coded as never versus ever smoker. Medication burden and comorbidity burden were defined as the total number of recorded concomitant medication categories and comorbidities, respectively. Stabilized inverse probability of treatment weights (IPTW) were then calculated and applied to generate a weighted pseudo-population with improved covariate balance between VV and MM/MV patients. Covariate balance before and after weighting was assessed using standardized mean differences, with an absolute standardized mean difference below 0.10 considered indicative of adequate balance. Balance diagnostics were summarized using Love plots. The association between MICA-129 VV status and overall survival was then evaluated using an IPTW-weighted Cox proportional hazards model. Hazard ratios, 95% confidence intervals, and two-sided p-values were reported.

Treatment-by-genotype interaction analysis

A treatment-by-genotype interaction analysis was conducted using a multivariable Cox proportional hazards model restricted to patients receiving *nintedanib* or *pirfenidone*. Untreated patients and therapy switchers were excluded from this analysis. MICA-129 genotype was analyzed under a recessive model, comparing VV carriers with MM/MV patients. The model included antifibrotic treatment, MICA-129 VV status, and the treatment × MICA-129 VV interaction term, with adjustment for age at diagnosis, sex, smoking status, medication burden, and comorbidity burden. Pirfenidone-treated patients and MM/MV genotypes were used as the reference categories. The interaction term was used to assess whether the VV-associated hazard ratio differed between nintedanib- and pirfenidone-treated patients. HRs, 95% CIs, and two-sided p-values were reported. The proportional hazards assumption was assessed using Schoenfeld residuals.

Adjusted longitudinal lung function analysis

Longitudinal changes in lung function were analyzed in the subgroup of patients treated with nintedanib. Changes in FVC% predicted and DLCO% predicted were calculated as the difference between follow-up and baseline values at 12, 24, and 36 months. Adjusted ΔFVC% predicted and ΔDLCO% predicted trajectories were estimated using linear mixed-effects models. Models included time point, MICA-129 genotype under the recessive VV model, and the genotype-by-time interaction, with adjustment for age at diagnosis, sex, smoking status, medication burden, and comorbidity burden. Estimated marginal means with 95% CIs were used to generate adjusted genotype-specific trajectories. The p-value shown in each figure was obtained from the type III F-test of the genotype-by-time interaction in the corresponding mixed-effects model. In addition, unadjusted Kruskal–Wallis tests were performed at each follow-up time point to compare raw changes from baseline between genotype groups.
